# *N*′-(2-Chloro­benzyl­idene)-4-nitro­benzohydrazide

**DOI:** 10.1107/S1600536811055589

**Published:** 2012-01-11

**Authors:** Chun-Bao Tang

**Affiliations:** a Department of Chemistry, Jiaying University, Meizhou 514015, People’s Republic of China

## Abstract

In the title compound, C_14_H_10_ClN_3_O_3_, the dihedral angle between the benzene rings is 6.64 (13)°. In the crystal, mol­ecules are linked through N—H⋯O hydrogen bonds, forming chains running along the *c* axis direction.

## Related literature

For general background to hydrazones, see: Rasras *et al.* (2010 ▶); Pyta *et al.* (2010 ▶); Angelusiu *et al.* (2010 ▶). For related structures, see: Fun *et al.* (2008 ▶); Singh & Singh (2010 ▶); Ahmad *et al.* (2010 ▶); Tang (2010 ▶, 2011 ▶). For reference bond-length data, see: Allen *et al.* (1987 ▶).
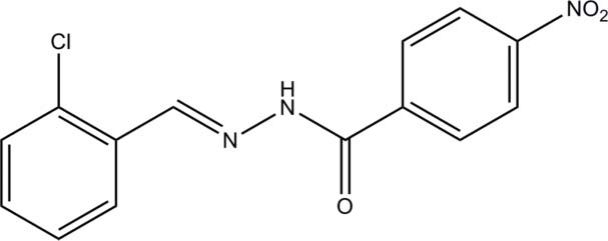


## Experimental

### 

#### Crystal data

C_14_H_10_ClN_3_O_3_
*M* *_r_* = 303.70
Monoclinic, 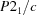

*a* = 11.2332 (18) Å
*b* = 13.3778 (18) Å
*c* = 8.9770 (16) Å
β = 90.408 (2)°
*V* = 1349.0 (4) Å^3^
*Z* = 4
Mo *K*α radiation
μ = 0.30 mm^−1^
*T* = 298 K
0.18 × 0.17 × 0.15 mm

#### Data collection

Bruker SMART CCD area-detector diffractometer
Absorption correction: multi-scan (*SADABS*; Sheldrick, 1996 ▶) *T*_min_ = 0.949, *T*_max_ = 0.957
10640 measured reflections
2931 independent reflections
1882 reflections with *I* > 2σ(*I*)
*R*_int_ = 0.045

#### Refinement

*R*[*F* ^2^ > 2σ(*F* ^2^)] = 0.051
*wR*(*F* ^2^) = 0.146
*S* = 1.03
2931 reflections
193 parameters
1 restraint
H atoms treated by a mixture of independent and constrained refinement
Δρ_max_ = 0.36 e Å^−3^
Δρ_min_ = −0.29 e Å^−3^

### 

Data collection: *SMART* (Bruker, 2002 ▶); cell refinement: *SAINT* (Bruker, 2002 ▶); data reduction: *SAINT*; program(s) used to solve structure: *SHELXS97* (Sheldrick, 2008 ▶); program(s) used to refine structure: *SHELXL97* (Sheldrick, 2008 ▶); molecular graphics: *SHELXTL* (Sheldrick, 2008 ▶); software used to prepare material for publication: *SHELXL97*.

## Supplementary Material

Crystal structure: contains datablock(s) global, I. DOI: 10.1107/S1600536811055589/su2353sup1.cif

Structure factors: contains datablock(s) I. DOI: 10.1107/S1600536811055589/su2353Isup2.hkl

Supplementary material file. DOI: 10.1107/S1600536811055589/su2353Isup3.cml

Additional supplementary materials:  crystallographic information; 3D view; checkCIF report

## Figures and Tables

**Table 1:** Hydrogen-bond geometry (Å, °)

*D*—H⋯*A*	*D*—H	H⋯*A*	*D*⋯*A*	*D*—H⋯*A*
N2—H2⋯O1^i^	0.89 (1)	2.06 (1)	2.915 (3)	160 (3)

Symmetry code: (i) 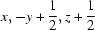.

**Fig. 1.**
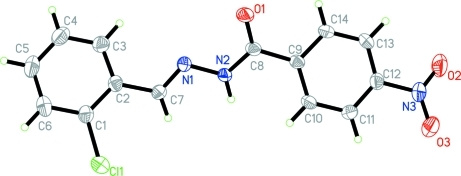
The molecular structure of the title molecule, showing the atom-numbering scheme. Displacement ellipsoids are drawn at the 30% probability level.

**Fig. 2.**
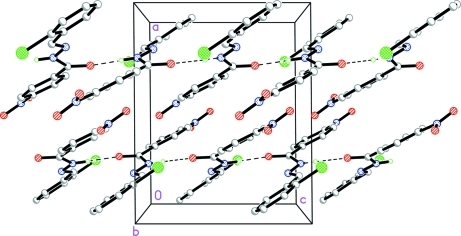
Crystal packing of the title compound, viewed along the b axis, with hydrogen bonds shown as dashed lines.

## supplementary crystallographic information

### Comment

Hydrazone compounds have received much attention in biological and structural chemistry in the last few years (Rasras *et al.*, 2010; Pyta *et al.*, 2010; Angelusiu *et al.*, 2010; Fun *et al.*, 2008; Singh & Singh, 2010; Ahmad *et al.*, 2010). As a continuation of our work on the structural study on such compounds (Tang, 2010, 2011), we reports herein on the crystal structure of the title new hydrazone compound.

In the molecule of the title compound (Fig. 1), the dihedral angle between the two benzene rings is 6.64 (13)°. Bond lengths in the compound are normal (Allen *et al.*, 1987) and comparable to those in the similar compounds (Tang, 2010; Tang, 2011).

In the crystal, molecules are linked through intermolecular N—H···O hydrogen bonds (Table 1), forming chains running along the *c* axis direction (Fig. 2).

### Experimental

2-Chlorobenzaldehyde (0.1 mmol, 14.1 mg) and 4-nitrobenzohydrazide (0.1 mmol, 18.2 mg) were dissolved in methanol (20 ml). The mixture was stirred at reflux for 10 min to give a clear yellow solution. Yellow needle-shaped crystals of the compound were formed by slow evaporation of the solvent over several days.

### Refinement

The amino H atom was located in a difference Fourier map and refined isotropically, with an N—H distance restraint of 0.90 (1) Å. C-bound H atoms were included in calculated positions and refined as riding atoms: C—H = 0.93 Å, with *U*_iso_(H) = 1.2*U*_eq_(C).

### Crystal data

**Table:**

C_14_H_10_ClN_3_O_3_	*F*(000) = 624
*M**_r_* = 303.70	*D*_x_ = 1.495 Mg m^−^^3^
Monoclinic, *P*2_1_/*c*	Mo *K*α radiation, λ = 0.71073 Å
*a* = 11.2332 (18) Å	Cell parameters from 2464 reflections
*b* = 13.3778 (18) Å	θ = 2.4–24.5°
*c* = 8.9770 (16) Å	µ = 0.30 mm^−^^1^
β = 90.408 (2)°	*T* = 298 K
*V* = 1349.0 (4) Å^3^	Cut from needle, yellow
*Z* = 4	0.18 × 0.17 × 0.15 mm

### Data collection

**Table:**

Bruker SMART CCD area-detector diffractometer	2931 independent reflections
Radiation source: fine-focus sealed tube	1882 reflections with *I* > 2σ(*I*)
graphite	*R*_int_ = 0.045
ω scans	θ_max_ = 27.0°, θ_min_ = 2.4°
Absorption correction: multi-scan (*SADABS*; Sheldrick, 1996)	*h* = −14→14
*T*_min_ = 0.949, *T*_max_ = 0.957	*k* = −14→17
10640 measured reflections	*l* = −11→11

### Refinement

**Table:**

Refinement on *F*^2^	Primary atom site location: structure-invariant direct methods
Least-squares matrix: full	Secondary atom site location: difference Fourier map
*R*[*F*^2^ > 2σ(*F*^2^)] = 0.051	Hydrogen site location: inferred from neighbouring sites
*wR*(*F*^2^) = 0.146	H atoms treated by a mixture of independent and constrained refinement
*S* = 1.03	*w* = 1/[σ^2^(*F*_o_^2^) + (0.0569*P*)^2^ + 0.5874*P*] where *P* = (*F*_o_^2^ + 2*F*_c_^2^)/3
2931 reflections	(Δ/σ)_max_ < 0.001
193 parameters	Δρ_max_ = 0.36 e Å^−^^3^
1 restraint	Δρ_min_ = −0.29 e Å^−^^3^

### Special details

**Table:**

Geometry. All esds (except the esd in the dihedral angle between two l.s. planes) are estimated using the full covariance matrix. The cell esds are taken into account individually in the estimation of esds in distances, angles and torsion angles; correlations between esds in cell parameters are only used when they are defined by crystal symmetry. An approximate (isotropic) treatment of cell esds is used for estimating esds involving l.s. planes.
Refinement. Refinement of F^2^ against ALL reflections. The weighted R-factor wR and goodness of fit S are based on F^2^, conventional R-factors R are based on F, with F set to zero for negative F^2^. The threshold expression of F^2^ > 2sigma(F^2^) is used only for calculating R-factors(gt) etc. and is not relevant to the choice of reflections for refinement. R-factors based on F^2^ are statistically about twice as large as those based on F, and R- factors based on ALL data will be even larger.

### Fractional atomic coordinates and isotropic or equivalent isotropic displacement parameters (Å^2^)

**Table:**

	*x*	*y*	*z*	*U*_iso_*/*U*_eq_	
Cl1	0.23505 (8)	0.62569 (6)	1.10065 (9)	0.0782 (3)	
H2	0.246 (3)	0.265 (2)	1.1045 (13)	0.080*	
N1	0.18950 (18)	0.33163 (14)	0.9198 (2)	0.0444 (5)	
N2	0.23517 (19)	0.25548 (14)	1.0072 (2)	0.0446 (5)	
N3	0.4488 (2)	−0.1478 (2)	1.3026 (3)	0.0667 (7)	
O1	0.26845 (19)	0.15983 (13)	0.80373 (18)	0.0620 (5)	
O2	0.4173 (3)	−0.23176 (19)	1.2688 (3)	0.1126 (10)	
O3	0.5111 (2)	−0.12928 (18)	1.4092 (3)	0.0902 (8)	
C1	0.1488 (2)	0.59971 (19)	0.9450 (3)	0.0513 (6)	
C2	0.1308 (2)	0.50090 (18)	0.8991 (3)	0.0452 (6)	
C3	0.0625 (2)	0.4872 (2)	0.7698 (3)	0.0553 (7)	
H3	0.0493	0.4227	0.7348	0.066*	
C4	0.0149 (3)	0.5657 (3)	0.6941 (3)	0.0679 (8)	
H4	−0.0312	0.5541	0.6094	0.082*	
C5	0.0344 (3)	0.6622 (2)	0.7416 (4)	0.0709 (9)	
H5	0.0020	0.7154	0.6885	0.085*	
C6	0.1015 (3)	0.6800 (2)	0.8673 (4)	0.0644 (8)	
H6	0.1149	0.7450	0.8998	0.077*	
C7	0.1807 (2)	0.41629 (18)	0.9816 (3)	0.0463 (6)	
H7	0.2061	0.4247	1.0796	0.056*	
C8	0.2731 (2)	0.17126 (17)	0.9389 (3)	0.0434 (6)	
C9	0.3213 (2)	0.09038 (17)	1.0373 (2)	0.0392 (5)	
C10	0.3769 (2)	0.10957 (18)	1.1718 (3)	0.0443 (6)	
H10	0.3845	0.1751	1.2052	0.053*	
C11	0.4216 (2)	0.03155 (19)	1.2576 (3)	0.0495 (6)	
H11	0.4605	0.0441	1.3474	0.059*	
C12	0.4069 (2)	−0.06443 (19)	1.2070 (3)	0.0479 (6)	
C13	0.3536 (2)	−0.08568 (19)	1.0728 (3)	0.0558 (7)	
H13	0.3456	−0.1514	1.0404	0.067*	
C14	0.3122 (2)	−0.00768 (19)	0.9871 (3)	0.0539 (7)	
H14	0.2777	−0.0207	0.8947	0.065*	

### Atomic displacement parameters (Å^2^)

**Table:**

	*U*^11^	*U*^22^	*U*^33^	*U*^12^	*U*^13^	*U*^23^
Cl1	0.1009 (6)	0.0532 (5)	0.0802 (6)	−0.0085 (4)	−0.0221 (5)	−0.0047 (4)
N1	0.0560 (12)	0.0374 (11)	0.0397 (11)	−0.0027 (9)	−0.0030 (9)	0.0046 (9)
N2	0.0662 (13)	0.0345 (11)	0.0329 (11)	−0.0010 (9)	−0.0045 (9)	−0.0007 (9)
N3	0.0637 (15)	0.0569 (16)	0.0792 (18)	0.0213 (12)	−0.0113 (13)	0.0006 (14)
O1	0.1089 (16)	0.0463 (10)	0.0308 (10)	−0.0018 (10)	−0.0020 (9)	−0.0018 (8)
O2	0.134 (2)	0.0514 (15)	0.152 (3)	0.0189 (14)	−0.0636 (19)	0.0076 (15)
O3	0.1053 (18)	0.0902 (18)	0.0747 (16)	0.0349 (14)	−0.0268 (14)	−0.0009 (13)
C1	0.0545 (14)	0.0475 (15)	0.0518 (15)	−0.0003 (12)	0.0045 (12)	0.0070 (12)
C2	0.0504 (13)	0.0428 (14)	0.0425 (14)	0.0001 (11)	0.0078 (11)	0.0052 (11)
C3	0.0590 (16)	0.0573 (17)	0.0496 (16)	0.0051 (13)	−0.0006 (12)	−0.0021 (13)
C4	0.0709 (19)	0.078 (2)	0.0548 (17)	0.0151 (16)	−0.0082 (14)	0.0057 (16)
C5	0.081 (2)	0.063 (2)	0.068 (2)	0.0191 (16)	0.0024 (17)	0.0202 (16)
C6	0.0742 (19)	0.0443 (16)	0.075 (2)	0.0044 (14)	0.0094 (16)	0.0094 (14)
C7	0.0579 (14)	0.0440 (14)	0.0369 (13)	−0.0017 (11)	−0.0019 (11)	0.0026 (11)
C8	0.0579 (14)	0.0369 (13)	0.0353 (13)	−0.0113 (11)	−0.0005 (10)	0.0003 (10)
C9	0.0476 (12)	0.0365 (12)	0.0337 (12)	−0.0051 (10)	0.0043 (10)	−0.0016 (10)
C10	0.0510 (13)	0.0369 (13)	0.0450 (14)	−0.0038 (10)	−0.0014 (11)	−0.0087 (11)
C11	0.0507 (14)	0.0530 (16)	0.0446 (14)	0.0034 (12)	−0.0080 (11)	−0.0057 (12)
C12	0.0468 (13)	0.0443 (14)	0.0526 (15)	0.0089 (11)	0.0004 (11)	0.0007 (12)
C13	0.0694 (17)	0.0355 (13)	0.0625 (18)	0.0044 (12)	−0.0095 (14)	−0.0119 (12)
C14	0.0749 (18)	0.0416 (14)	0.0450 (14)	−0.0001 (13)	−0.0130 (13)	−0.0117 (12)

### Geometric parameters (Å, °)

**Table:**

Cl1—C1	1.729 (3)	C4—H4	0.9300
N1—C7	1.265 (3)	C5—C6	1.373 (4)
N1—N2	1.382 (3)	C5—H5	0.9300
N2—C8	1.353 (3)	C6—H6	0.9300
N2—H2	0.891 (10)	C7—H7	0.9300
N3—O3	1.207 (3)	C8—C9	1.495 (3)
N3—O2	1.216 (3)	C9—C10	1.380 (3)
N3—C12	1.482 (3)	C9—C14	1.390 (3)
O1—C8	1.224 (3)	C10—C11	1.389 (3)
C1—C6	1.385 (4)	C10—H10	0.9300
C1—C2	1.399 (3)	C11—C12	1.372 (3)
C2—C3	1.399 (3)	C11—H11	0.9300
C2—C7	1.462 (3)	C12—C13	1.371 (3)
C3—C4	1.358 (4)	C13—C14	1.375 (4)
C3—H3	0.9300	C13—H13	0.9300
C4—C5	1.377 (5)	C14—H14	0.9300
			
C7—N1—N2	116.14 (19)	N1—C7—C2	120.1 (2)
C8—N2—N1	118.26 (19)	N1—C7—H7	119.9
C8—N2—H2	122 (2)	C2—C7—H7	119.9
N1—N2—H2	120 (2)	O1—C8—N2	122.8 (2)
O3—N3—O2	123.6 (3)	O1—C8—C9	120.5 (2)
O3—N3—C12	119.0 (3)	N2—C8—C9	116.7 (2)
O2—N3—C12	117.4 (3)	C10—C9—C14	119.4 (2)
C6—C1—C2	122.0 (3)	C10—C9—C8	122.8 (2)
C6—C1—Cl1	117.5 (2)	C14—C9—C8	117.8 (2)
C2—C1—Cl1	120.5 (2)	C9—C10—C11	120.3 (2)
C1—C2—C3	116.5 (2)	C9—C10—H10	119.8
C1—C2—C7	121.9 (2)	C11—C10—H10	119.8
C3—C2—C7	121.6 (2)	C12—C11—C10	118.5 (2)
C4—C3—C2	121.7 (3)	C12—C11—H11	120.7
C4—C3—H3	119.2	C10—C11—H11	120.7
C2—C3—H3	119.2	C13—C12—C11	122.4 (2)
C3—C4—C5	120.6 (3)	C13—C12—N3	119.2 (2)
C3—C4—H4	119.7	C11—C12—N3	118.4 (2)
C5—C4—H4	119.7	C12—C13—C14	118.5 (2)
C6—C5—C4	120.1 (3)	C12—C13—H13	120.7
C6—C5—H5	119.9	C14—C13—H13	120.7
C4—C5—H5	119.9	C13—C14—C9	120.7 (2)
C5—C6—C1	119.1 (3)	C13—C14—H14	119.6
C5—C6—H6	120.4	C9—C14—H14	119.6
C1—C6—H6	120.4		

### Hydrogen-bond geometry (Å, °)

**Table:**

*D*—H···*A*	*D*—H	H···*A*	*D*···*A*	*D*—H···*A*
N2—H2···O1^i^	0.89 (1)	2.06 (1)	2.915 (3)	160 (3)

Symmetry codes: (i) *x*, −*y*+1/2, *z*+1/2.
